# Platelet to lymphocyte ratio in the prediction of adverse outcomes after acute coronary syndrome: a meta-analysis

**DOI:** 10.1038/srep40426

**Published:** 2017-01-10

**Authors:** Wenzhang Li, Qianqian Liu, Yin Tang

**Affiliations:** 1 Department of Cardiology, First Affiliated Hospital of Chengdu Medical College, Chengdu, Sichuan, China; 2 Department of Respiratory Diseases, Chengdu Municipal First People’s Hospital, Chengdu, Sichuan, China; 3 State Key Laboratory of Oral Disease, West China School & Hospital of Stomotology, Sichuan University, Chengdu, Sichuan, China

## Abstract

Recent studies have shown platelet to lymphocyte ratio (PLR) to be a potential inflammatory marker in cardiovascular diseases. We performed a meta-analysis to systematically evaluate the prognostic role of PLR in acute coronary syndrome (ACS). A comprehensive literature search up to May 18, 2016 was conducted from PUBMED, EMBASE and Web of science to identify related studies. The risk ratio (RR) with 95% confidence interval (CI) was extracted or calculated for effect estimates. Totally ten studies involving 8932 patients diagnosed with ACS were included in our research. We demonstrated that patients with higher PLR level had significantly higher risk of in-hospital adverse outcomes (RR = 2.24, 95%CI = 1.81–2.77) and long-term adverse outcomes (RR = 2.32, 95%CI = 1.64–3.28). Sensitivity analyses confirmed the stability of our results. We didn’t detect significant publication bias by Begg’s and Egger’s test (p > 0.05). In conclusion, our meta-analysis revealed that PLR is promising biomarker in predicting worse prognosis in ACS patients. The results should be validated by future large-scale, standard investigations.

Acute coronary syndrome (ACS), characterized by unstable atherosclerotic lesions, is the main cause of death from coronary heart diseases^1^. Other major adverse cardiovascular event (MACE) following ACS, such as re-infarction and recurrent ischaemia, also pose heavy burden on health-care resources^2^. Even with the advent of coronary intervention techniques and progress in medication, the prognosis of ACS is still not satisfactory^3^. Thus, it is of great significance to identify high-risk ACS patients who require more intensive control of risk factors, more aggressive therapy and more close follow-up.

The interaction of inflammation and thrombosis is the critical factor in the pathogenesis of ACS^4^. As a reflection of excess inflammatory status and thrombotic activity, elevated peripheral blood platelet count is regarded to be a valuable predictor of adverse cardiovascular outcomes. And multiple researches have demonstrated this speculation^5^,^6^. On the other hand, decreased lymphocyte count is also related to worse cardiovascular prognosis, which may be partly explained by the role of lymphocyte in protection of plaque stability^7^. In this context, a novel marker, platelet to lymphocyte ratio (PLR), seemed to be a potential indicator in ACS prognosis.

Recently, the prognostic importance of PLR has been investigated by several studies. Zhou *et al*. demonstrated that PLR was positively associated with the Global Registry of Acute Coronary Events (GRACE) risk score and can improve its the predictive power for long-term cardiovascular events in patients with ACS^8^. Temiz *et al*. reported that PLR was an independent predictor of in-hospital cardiovascular mortality in patients with ST-elevated acute myocardial infarction^9^. Ugur *et al*. found that there was a significant association between high PLR levels and the adjusted risk of 6-month all-cause deaths in ST elevation myocardial infarction underwent primary coronary intervention, while with no difference in in-hospital mortality among patients with different PLR levels^10^. Although most studies demonstrated the positive association between PRL and ACS adverse outcomes, there are still some discrepancies. Hence, we performed this meta-analysis to investigate the role of PLR in evaluating the prognosis of ACS, focusing on in-hospital and long-term adverse outcomes respectively. This study was reported according to the Preferred Reporting Items for Systematic Reviews and Meta-Analyses (PRISMA)^11^.

## Methods

### Search strategy

We conducted a comprehensive computer search through the following databases from their inception until May 18, 2016: PUBMED, EMBASE and Web of Science. No language restriction was applied. The following terms was used to identify related articles: ‘platelet’ in combination with ‘lymphocyte’, and in combination with ‘acute coronary syndrome’ or ‘myocardial infarction’ or ‘myocardial ischaemia’ or ‘unstable angina’. The reference lists of all included studies were also screened to check for relevant articles.

### Inclusion and exclusion criteria

The inclusion criteria were listed as follows: (1) All patients were diagnosed as ACS, including acute ST elevation myocardial infarction, acute non-ST elevation myocardial infarction and unstable angina^8^. (2) Blood samples for PLR determination were taken at admission. (3) Outcomes were death, re-infarction, postinfarction angina, recurrent ischaemia or need for revascularization^12^. Patients were followed up during hospitalization (in-hospital outcomes) or for at least six month (long-term outcomes) (4) Sufficient data were reported to estimate the relative risk (RR) with 95% confidence interval (CI). Exclusion criterion was duplicates of previous publications.

### Data extraction and quality assessment

Two investigators (Qianqian Liu and Wenzhang Li) were responsible for data extraction and quality assessment. The following information were obtained: title, journal, author, language, year of publication, study design, country, sample size, mean age, gender, diagnosis, outcomes, adjusted or unadjusted risk estimates. The Newcastle-Ottawa scale (NOS) was used to assess the methodological quality of included studies, which is composed of three aspects (selection, comparability and outcome), with 9 being the highest score. We considered studies with a NOS score more than or equal to seven to be of high quality. Discrepancies were discussed by the two reviewers.

### Statistical analysis

We performed meta-analysis on association between PLR and ACS prognosis using RR estimates and 95% CIs on a natural logarithmic scale. Compared with crude RR, the adjusted RR were preferred when available. We transformed adjusted odds ratio (OR) to RR when necessary with methods provided by Zhang *et al*.^13^. Statistical heterogeneity was tested by Cochran’s Q statistic and I^2^ tests. Fixed effect model was used if the *p* > 0.10 and I^2^ < 50% otherwise the random effect model was adopted^14^,^15^. In order to confirm the robust of pooled results, we performed a sensitivity analysis by excluding each study one by one. Begg’s and Egger’s tests were conducted to identify possible publication bias^16^. Statistical analyses were done with STATA version 12.0 (StataCorp, College Station, Texas), using metan program. *P* values were two-sided.

## Results

### Study characteristics

**Figure 1:**
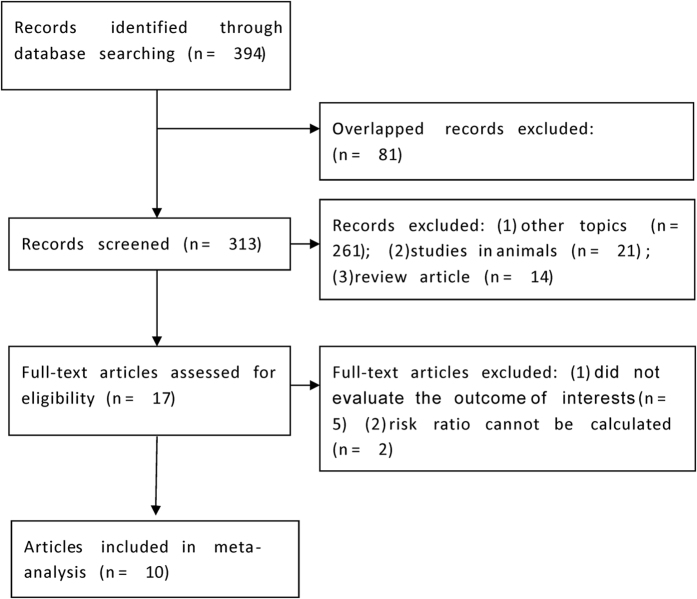
Flow diagram of included studies.

Totally ten studies involving 8932 patients diagnosed with ACS were included in our final meta-analyses (Fig. 1)^8^,^9^,^10^,^17^,^18^,^19^,^20^,^21^,^22^,^23^. The characteristics of eligible studies are listed in Table 1. They were published between 2012 and 2016, and conducted at hospitals from Europe (seven in Turkey, one in Poland), Asia (one in China), and America (one in USA) respectively. The sample size of each study ranged from 304 to 2230. The cut-off value for PLR ranged from 116 to 174.9 in eight studies. While the other two studies didn’t present specific cut-off values and they divided patients into groups according to the PLR tertiles (comparing the third tertile versus others). The follow-up duration of long-term adverse outcomes was between one to sex years. Among all these studies, seven were prospective cohort, while the other three were retrospective cohort. All of ten studies were classified to be high-quality, according to NOS score.

### Data synthesis

Eight studies, including 6083 patients were evaluated for in-hospital adverse outcomes while six study, involving 6253 patients were assessed for long-term adverse outcomes. The between-study heterogeneity was insignificant in in-hospital adverse outcomes (*p* = 0.180), while obvious in long-term adverse outcomes (*p* < 0.001).

**Figure 2:**
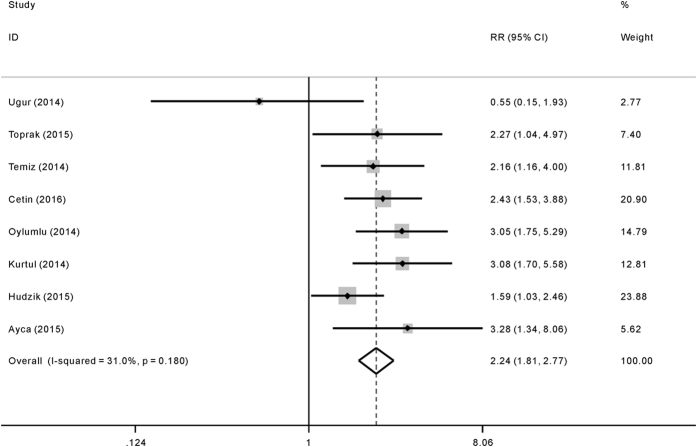
Forrest plot of risk ratio (RR) for the association of platelet to lymphocyte ratio (PLR) with in-hospital adverse outcomes after acute coronary syndrome (ACS).

**Figure 3:**
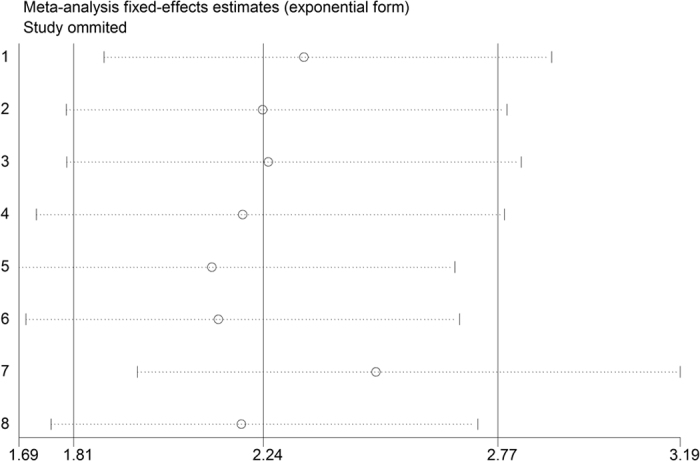
Sensitivity analysis of risk ratio (RR) for the association of platelet to lymphocyte ratio (PLR) with in-hospital adverse outcomes after acute coronary syndrome (ACS).

Compared with lower PLR level, patients with higher PLR level had significantly higher risk of in-hospital adverse outcomes (RR = 2.24; 95%CI = 1.81–2.77), see Fig. 2. The subsequent subgroup analyses based on different mean age, sample size, region, outcome and confounding factors adjustment were presented in Table 2, which demonstrated the robust poor prognosis among patients with higher PLR level (*p* < 0.05). Sensitivity analysis was shown in Fig. 3. After excluding each study one by one, the corresponding pooled RRs were not obviously changed.

**Figure 4:**
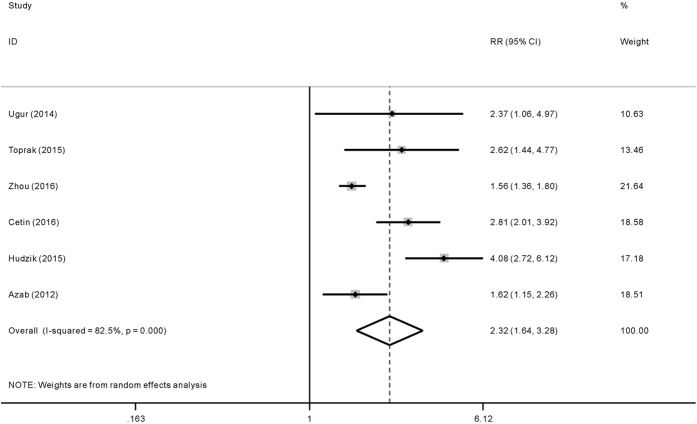
Forrest plot of risk ratio (RR) for the association of platelet to lymphocyte ratio (PLR) with long-term adverse outcomes after acute coronary syndrome (ACS).

**Figure 5:**
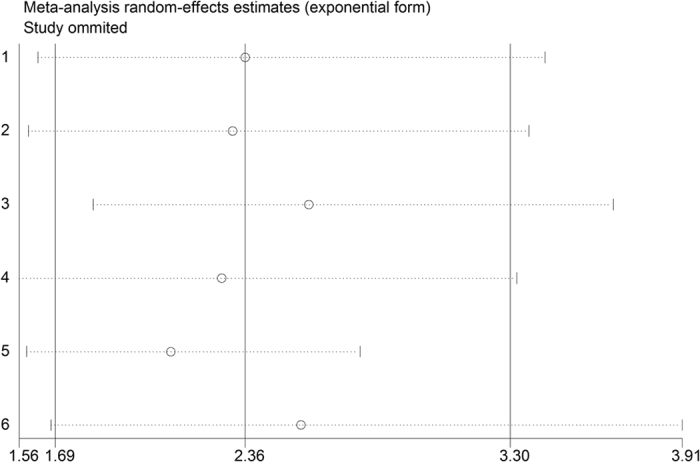
Sensitivity analysis of risk ratio (RR) for the association of platelet to lymphocyte ratio (PLR) with long-term adverse outcomes after acute coronary syndrome (ACS).

The pooled RRs of long-term adverse outcomes were 2.32 (95%CI: 1.64–3.28) for patients with higher PLR level, see Fig. 4. The risk effect of higher PLR level on long-term adverse outcomes was not significantly influenced by age, sample size, region, outcome and confounding factors adjustment (p < 0.05), as is shown in Table 2. It is worth mentioning that the between-study heterogeneity disappeared during the subgroup analyses according to region, which may partly account for the source of heterogeneity. To examine the influence of individual study upon the results, sensitivity analysis was conducted and we arrived almost the same results (Fig. 5).

### Publication bias

**Figure 6:**
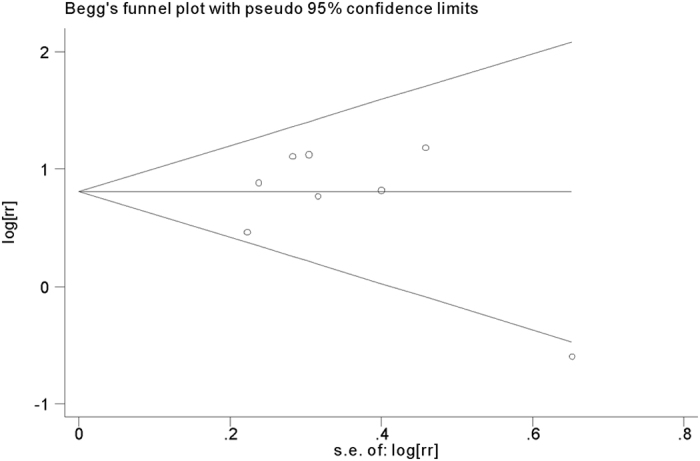
Funnel plot of selected studies for the association of platelet to lymphocyte ratio (PLR) with in-hospital adverse outcomes after acute coronary syndrome (ACS).

**Figure 7:**
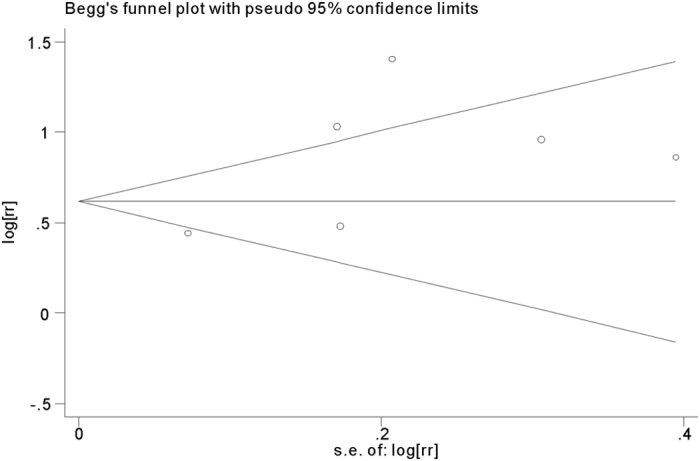
Funnel plot of selected studies for the association of platelet to lymphocyte ratio (PLR) with long-term adverse outcomes after acute coronary syndrome (ACS).

Begg’s funnel plot and Egger’s test were performed to evaluate the publication bias of the included studies. The funnel plot was symmetric for both in-hospital and long-term adverse outcomes (Figs 6 and 7). Both Begg’s and Egger’s test showed lack of publication bias (*p* = 0.662 and *p* = 0.121).

## Discussion

PLR, as a systematic inflammatory response indicator, was initially introduced into clinical practice to improve the prognosis prediction of oncologic disorders, such as periampullary cancer, esophageal cancer and gastric cancer *et al*.^24^,^25^,^26^,^27^. After that this marker was found to be associated with inflammation in cardiovascular diseases and positively correlated with SYNTAX score, GRACE risk and no-reflow phenomenon in ACS9^20^,^28^,^29^. Most recently a series of studies have investigated the predictive importance of PLR in the adverse outcomes among ACS and reported inconsistent results^9^,^10^,^22^,^29^. We conducted this meta-analysis to comprehensively understand the prognostic role of PLR in ACS. Our results demonstrated that patients with higher PLR level had 2.24-fold higher risk of in-hospital adverse outcomes and 2.32-fold higher risk of long-term adverse outcomes. The reliability and stability of our results were confirmed by publication bias and sensitivity analyses.

The between-study heterogeneity was insignificant when analyzing in-hospital adverse outcomes, which indicated the reliability of our pooling results. However, there was obvious heterogeneity when pooling the data regarding PLR and long-term adverse outcomes. Then we did subgroup analyses to search for potential factors that lead to heterogeneity. Our subgroup analyses demonstrated that the risk effects of higher PLR level in ACS adverse outcomes were not significantly influenced by potential confounding factors, such as age, sample size, region, outcome and risk factors adjustment. It should be pointed out that the between-study heterogeneity disappeared during the subgroup analyses according to region. We postulate that region may partly explained the observed heterogeneity and it might be ascribed to the following factors. First, as genetic variants have already be demonstrated to be related to hematological traits based on a series of genetic association studies, we believe that the predictive significance of PLR may also be different because of ethnicitic genotypic diversity^30^. And PLR also showed significant ethnic variability in other diseases, such as non-small cell lung cancer^31^. Second, some environmental factors, such as season variation, dietary habit and so on, which were reported to be confounding factors of ACS prognosis may also account for different results from different regional areas^32^,^33^. However those are only speculations before more evidences were obtained.

The underlying mechanisms of the association between higher PLR and worse prognosis seem to be multifactorial. Firstly, higher platelet counts serve to be both a result and a precipitating factor of inflammatory response. It is reported that megakaryocyte could be stimulated by several inflammatory mediators and presented accelerated proliferation and platelet-production^34^. On the other hand, platelets can release thromboxanes and other mediators, promote the adhesion and transmigration of monocytes, which may cause increased inflammation and weakened plaque stability, then promote the progression of atherosclerosis^35^,^36^,^37^,^38^. Additionally the procoagulant role of platelets in the process of homeostasis and thrombosis may also contribute to the progression of the arterial thrombi^39^. On the contrary, the elevated lymphocyte in ischaemic and reperfused myocardium can regulate mononuclear cell phenotype transformation and induce tissue inhibitor of matrix metalloproteinase-1 production, thereby increasing plaque stability in ACS patients^7^. Beyond that lymphopenia was speculated to represent depressed immune response to excess cortisol production during physiological stress, which may lead to worse clinical outcomes^40^. And it has been reported that lymphocytopenia was independently related to adverse cardiac events, advanced heart failure, as well as reduced hemodynamics and aerobic capacity^41^,^42^,^43^,^44^,^45^. Taken all above together, as a combined marker of thrombocytosis and lymphocytopenia, PLR may be promising in ACS prognosis prediction.

The significance of PLR in ACS prognosis prediction can also be validated through its association with other predictors. Ayhan *et al*. found that patients with higher PLR levels exhibited higher peak CK-MB, creatinine, Killip class, admission glucose, and anemia in their study^46^,^47^,^48^. Zhou *et al*. showed a positive association between GRACE risk score and PLR^8^. The PLR was also reported to be significantly correlated with other inflammatory markers such as C-reactive protein (CRP) and fibrinogen, which have been demonstrated to have predictive and prognostic significance in cardiovascular diseases^39^. Up till now, we didn’t find systematic research comparing the significance of those predictors. Which independent or joint predictor is the best of choice is still a matter of debate. However, with easy and rapid availability and relative low costs, PLR is undoubtedly a promising marker which need to be further explored.

It is interesting that, in some cases, thrombocytopenia may also indicate increased risk of ischemic events, which seems to be a paradox to our results^49^,^50^. However, further studies found that, in the population of ischemic patients, it was the use of intra-aortic balloon pump (IABP), but not thrombocytopenia per se, serves as a possible primary cause of adverse ischemic events^49^,^50^. It reminds us that when we use PLR to predict the prognosis of ACS, we should make our judgment according to each specific matter considering possible confounding factors.

We had to admit that although there is good reason for PLR to be used as a predictive markers in worse prognosis among ACS patients, our research didn’t provide direct evidence of independent association between them. Other blood cell counts parameters, which are accompanied by or interfere with the change of PLR, were also demonstrated to be associated with adverse cardiovascular outcomes. For example, elevated red blood cell count, which is usually accompanied with activation, adhesiveness, and aggregation of platelet, can bring about increased blood viscosity and then further promote the genesis of ischaemic heart disease^51^. Puddu *et al*. reported that increased red blood cell count is independently associated with risk of cardiovascular atherosclerotic events in their study of 6 years follow-up^52^. On the other hand, anemia were also a concomitant of platelet activation in ACS patients as the consequence of chronic inflammatory response^53^. Several researches reported anemia was related to death as well as MACE and which was inferred to be the result of oxygen supply and demand imbalance, rennin-angiotensin-aldosterone activation and sypathetic nervous system activation^53^,^54^,^55^. In a word, whether or not PLR serves as the causal factor for adverse outcomes in ACS patients need to be verified.

Some limitations in our meta-analysis should be addressed. First of all, most studies included in our research were conducted in European countries, which may weaken the representative of results. Secondly, some studies did not adjust for potential confounders, which may limit the accuracy of the risk estimates. Thirdly, the cut-off point of PLR in two studies was not described, which limit the application of our research. Finally, some statistical limitations in our research should also be pointed out. No matter we use Egger’s or Begg’s test, we had to mention that the power to detect publication bias is low with a small number of included studies^56^. What’s more, Cochran’s Q and I^2^, which were adopted to detect between-study heterogeneity in our study, can be misleading in meta-analyses if the appropriate reference Q value is not considered^57^.

In conclusion, our meta-analysis revealed that PLR is promising biomarker in predicting both in-hospital and long-term worse prognosis in ACS patients. The results should be validated by future large-scale, standard investigations.

## Additional Information

**How to cite this article**: Li, W. *et al*. Platelet to lymphocyte ratio in the prediction of adverse outcomes after acute coronary syndrome: a meta-analysis. *Sci. Rep.* **7**, 40426; doi: 10.1038/srep40426 (2017).

**Publisher's note:** Springer Nature remains neutral with regard to jurisdictional claims in published maps and institutional affiliations.

## Figures and Tables

**Table 1:** Characteristics of studies included in meta-analysis.

Author (year)	Study design	Country	Sample size	Mean age (years)	Male (%)	Diagnosis	Adverse outcomes	RR^a^	RR^b^	Mean follow-up (months)	Cut-off	NOS
Ugur^10^	prospective cohort	Turkey	639	56	84.80	STEMI	Death	RR = 0.55 (0.15–1.93)	RR = 2.37 (1.06–4.97)^d^	6	174.9	8
Toprak^17^	prospective cohort	Turkey	304	60	80.90	STEMI	MACE	RR = 2.27 (1.04–4.97)	RR = 2.62 (1.44–4.77)	24	141	7
Zhou^8^	prospective cohort	China	2230	59	58.10	ACS	MACE	NA	RR = 1.56 (1.36–1.80)	72	170	7
Temiz^9^	retrospective cohort	Turkey	636	62	82.50	STEMI	Death	RR = 2.16 (1.16–4.0)^c^	NA	NA	144	9
Cetin^16^	prospective cohort	Turkey	1938	60	66.40	STEMI	MACE	RR = 2.43 (1.53–3.88)	RR = 2.81 (2.01–3.92)	31.6	NA	8
Oylumlu^19^	retrospective cohort	Turkey	587	62	68.40	ACS	Death	RR = 3.05 (1.75–5.29)	NA	NA	NA	8
Kurtul^20^	prospective cohort	Turkey	1016	61	71.90	ACS	Death	RR = 3.08 (1.70–5.58)	NA	NA	116	7
Hudzik^21^	prospective cohort	Poland	523	64	41.50	STEMI	Death	RR = 1.59 (1.03–2.46)	RR = 4.08 (2.72–6.12)	12	124	7
Azab^22^	prospective cohort	USA	619	61–68	68.50	NSTEMI	Death	NA	RR = 1.62 (1.15–2.26)^e^	48	176	8
Ayca^23^	retrospective cohort	Turkey	440	56–59	66.80	AMI	Death	RR = 3.28 (1.34–8.06)	NA	NA	137	8

**Abbreviations:** RR, risk ratio; NOS, Newcastle-Ottawa scale; STEMI, ST elevated myocardial infarction; MACE: non-fatal major adverse cardiovasculara events; ACS, acute coronary syndrome; NA: not available; NSTEMI, non-ST elevated myocardial infarction; AMI, acute myocardial infarction; PLR: platelet to lymphocyte ratio; GRE, glomerular filtration rate; TIMI, thrombolysis in mycocardial infarction.

^a^RR of PLR on in-hospital adverse outcomes.

^b^RR of PLR on long-term adverse outcomes (more than one month).

^c^Age, no thromolytic treatment, GFR.

^d^Age, sex, hypertension, left ventricular ejection fraction, anemia, post TMID flow, Killip class, GFR, three-vessel disease.

^e^GRACE score, use of aspirin or clopidogrel, prior coronary bypass surgery, diabetes mellitus, use of statin, end stage renal disease, prior cerebrovascular events.

**Table 2:** Subgroup analysis.

Subgroup	parameter	in-hospital adverse outcomes					long-term adverse outcomes				
		No. of studies	No. of patients	RR (95%CI)	*p* value	*p* for heterogeneity	No.of studies	No. of patients	RR (95%CI)	*p* value	*p* for heterogeneity
mean age	≤60	4	3321	2.24 (1.58–3.19)	<0.001	0.141	4	5111	2.19 (1.47–3.26)	<0.001	0.005
	>60	4	2762	2.24 (1.72–2.93)	<0.001	0.196	2	1142	2.55 (1.03–6.31)	0.042	0.001
sample Size	≤500	2	744	2.66 (1.48–4.80)	0.001	0.094	1	304	2.62 (1.44–4.77)	0.002	NA
	>500	6	5339	2.19 (1.74–2.74)	<0.001	0.544	5	5949	2.28 (1.55–3.35)	<0.001	<0.001
region	Europe	8	6083	2.24 (1.81–2.77)	<0.001	0.180	4	3404	3.08 (2.46–3.86)	<0.001	0.414
	non-Europe	0	0	NA	NA	NA	2	2849	1.57 (1.38–1.79)	<0.001	0.840
outcomes	Death	6	3841	2.19 (1.70–2.81)	<0.001	0.075	3	1781	2.50 (1.30–4.81)	0.006	0.003
	MACE	2	2242	2.39 (1.60–3.56)	<0.001	0.883	3	4472	2.17 (1.37–3.45)	0.001	0.002
adjustment	yes	1	636	2.16 (1.16–4.01)	0.015	NA	2	1258	1.72 (1.26–2.35)	0.001	0.376
	no	7	5447	2.25 (1.80–2.83)	<0.001	0.119	4	4995	1.87 (1.66–2.11)	<0.001	<0.001

**Abbreviations:** RR, risk ratio; NOS; Newcastle-Ottawa scale; STEMI, ST elevated myocardial infarction; MACE: non-fatal major adverse cardiovascular events; ACS, acute coronary syndrome; NA: not available; NSTEMI, non-ST elevated myocardial infarction; AMI, acute myocardial infarction; PLR: platelet to lymphocyte ratio; GRE, glomerular filtration rate; TIMI, thrombolysis in mycocardial infarction.
